# Primary Sjögren's Syndrome Accompanied by Clinical Features of TAFRO Syndrome

**DOI:** 10.1155/2020/8872774

**Published:** 2020-09-17

**Authors:** Eiji Suzuki, Takuya Ichimura, Satoru Kimura, Takashi Kanno, Kiyoshi Migita

**Affiliations:** ^1^Department of Rheumatology, Ohta-Nishinouchi Hospital, 2-5-20, Nishinouchi, Koriyama City, Fukushima 963-8558, Japan; ^2^Department of Hematology, Ohta-Nishinouchi Hospital, 2-5-20, Nishinouchi, Koriyama City, Fukushima 963-8558, Japan; ^3^Department of Rheumatology, Fukushima Medical University School of Medicine, 1, Hikarigaoka, Fukushima City, Fukushima 960-1295, Japan

## Abstract

Sjögren's syndrome (SS) is associated with not only sicca symptoms but also various symptoms caused by extraglandular manifestation. The pathophysiology and comorbidities of TAFRO syndrome (thrombocytopenia, anasarca, fever, reticulin fibrosis, and organomegaly), which is thought to be a variant of multicentric Castleman's disease, are not fully understood, and there are few data on the effectiveness of treatments. We report a patient of SS with TAFRO syndrome-like clinical features. A 52-year-old woman was admitted to our hospital because of abdominal distension. Laboratory data showed thrombocytopenia, and image findings showed massive ascites without evidence of malignant disease as confirmed by cytology. She was diagnosed with SS based on dysfunction of salivary secretion and positivity for anti-Ro/SS-A and La/SS-B antibodies, accompanied by clinical features of TAFRO syndrome based on the presence of anasarca and thrombocytopenia. High-dose corticosteroid for inflammation, anasarca, and thrombocytopenia was not effective. Cyclosporine was administered next, but anasarca and thrombocytopenia did not immediately improve until tolvaptan and eltrombopag were added. Although tolvaptan and eltrombopag were used for only a few months, the patient maintained a good condition with cyclosporine and low-dose prednisolone. In SS patients, activation of antigen-specific T lymphocytes is thought to be an important trigger that accelerates the immune response and is followed by hypercytokinemia. Therefore, using cyclosporine to suppress the activity of T lymphocytes is a reasonable treatment for SS accompanied with TAFRO syndrome-like pathophysiology. It might also be useful to administer tolvaptan or eltrombopag before the effects of immunosuppressants appear. If refractory inflammation with anasarca, thrombocytopenia, or lymphadenopathy is observed in an SS patient, complications with TAFRO syndrome-like pathophysiology should be considered.

## 1. Introduction

Sjögren's syndrome (SS) is an autoimmune disease characterized by lymphocytic infiltration into the lacrimal and salivary glands leading to symptomatic dry eyes and mouth and B-lymphocyte hyperreactivity [1]. The total number of patients with SS across Japan in 2011 was estimated at 68,483, and the prevalence was 0.05% based on surveys of the epidemiology of SS in Japan [2]. Decreased functioning of the exocrine glands leads to a combination of dry eye and dry mouth symptoms [3]. However, many organs other than the exocrine glands are affected in SS patients: skin, joints, lung, heart, gastrointestinal tract including the pancreas and liver, kidneys, bladder, the gynecological system, and both the peripheral and central nervous systems [4]. The pathogenesis of SS is thought to be a multistep process triggered by environmental factors in a genetically predisposed individual. As a result of this process, activated T cells produce various cytokines and induce chronic inflammation, and an antigen-nonspecific immune response might also be established [5,6].

TAFRO syndrome, first reported by Takai et al. in 2010 [7], is a systemic inflammatory disease characterized by thrombocytopenia, anasarca (edema, pleural effusion, and ascites), fever, reticulin myelofibrosis (or renal insufficiency), and organomegaly (hepatosplenomegaly and lymphadenopathy) [8]. Masaki et al. proposed diagnostic criteria and a disease severity classification for TAFRO syndrome in 2015 [8]. TAFRO syndrome was initially considered a variant of idiopathic multicentric Castleman's disease (MCD) because some of the histopathological features of TAFRO syndrome are similar to those of mixed-type MCD. However, thrombocytopenia and anasarca cannot be explained solely by the hyper-interleukin- (IL-) 6 syndrome of MCD [8]. The etiology and pathophysiology of TAFRO syndrome have not yet been clarified.

In this report, we describe a patient who was diagnosed with primary SS based on dysfunction of salivary secretion and positivity for anti-Ro/SS-A and anti-La/SS-B antibodies. These symptoms were accompanied by clinical features of TAFRO syndrome, including refractory pleural effusion, ascites, edema, and severe thrombocytopenia. She was initially treated with a corticosteroid, but her condition did not improve. After adding cyclosporine, her condition eventually improved. Additionally, eltrombopag and tolvaptan were effective as supportive care until the effects of cyclosporine appeared.

## 2. Case Presentation

**Figure 1:**
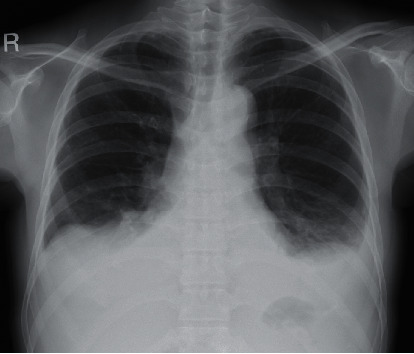
Chest X-ray on admission showing bilateral dullness of the costophrenic angles.

**Figure 2:**
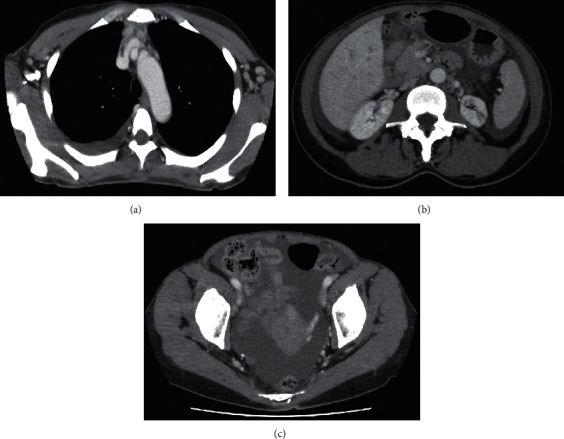
Computed tomography revealing enlargement of the axillary lymph nodes and right pleural effusion (a) and ascites around the liver and spleen (b) and in the pelvic cavity (c).

**Figure 3:**
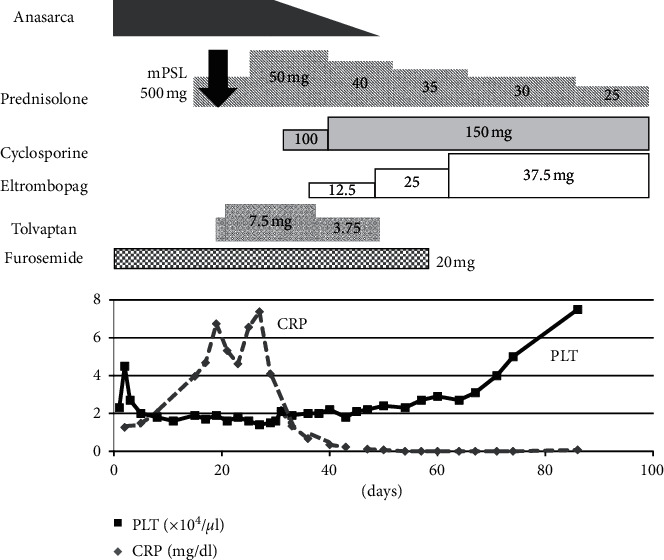
Clinical course of treatment and response of the patient. CRP, C-reactive protein; mPSL, methylprednisolone; PLT, platelet.

A 52-year-old woman was admitted to the Department of Obstetrics and Gynecology of Ohta-Nishinouchi Hospital because of abdominal distension. One month prior, she had visited a neighboring clinic because she felt abdominal fullness. She was shown to have a low platelet count of 27,000/*μ*L and ascites. Computed tomography and magnetic resonance imaging examinations were performed; however, no malignancies were observed. Cytology of the ascites did not reveal malignant cells. Because autoimmune disease was suspected, she was transferred to our department. She had experienced spondylolisthesis in her twenties and was diagnosed with nontuberculous mycobacterial infection at age 51, for which she took medication. At the time of transfer, her body weight was 58 kg, which was 10 kg heavier than usual. Her body temperature was 36.6°C, blood pressure was 112/70 mmHg, and pulse rate was 80 beats/minute. Several small-sized, nontender lymph nodes were palpable in her left neck. Her abdomen was slightly distended without tenderness. Her hands and fingers were slightly edematous. No skin rashes were observed. The laboratory findings were as follows: platelet count of 27 × 10^3^/*μ*L; anti-nuclear antibody positive at a titer of 1 : 320 (speckled and cytoplasmic pattern); anti-Ro/SS-A antibody and anti-La/SS-B antibody positive at titers of 1 : 32 and 1 : 1, respectively (Ouchterlony method); human herpesvirus 8 DNA negative; and slight positivity for IL-6 and vascular endothelial growth factor (VEGF) at 3.01 pg/mL and 42.1 pg/mL, respectively (Table 1). Chest X-ray showed bilateral dullness of the costophrenic angles and no enlargement of the heart shadow (Figure 1). Computed tomography showed enlargement of the axillary lymph nodes, splenomegaly, and a mild volume of ascites around the liver and spleen and in the pelvic cavity (Figure 2). We found no increases in megakaryocytes or reticulin fibrosis in the bone marrow specimens. She was diagnosed with SS using the revised criteria for the diagnosis of SS issued by the Japanese Ministry of Health [9]. The patient fulfilled the criteria of dysfunction of salivary secretion based on the results of the Saxon test (0.2 g/2 minutes) and salivary scintigraphy and positivity for anti-Ro/SS-A and anti-La/SS-B antibodies; however, the presence of anasarca and severe thrombocytopenia did not coincide with the pathophysiology of SS. She also fulfilled the proposed diagnostic criteria for all three of the major categories of TAFRO syndrome (anasarca, thrombocytopenia, and systemic inflammation), as well as two of the minor categories (mild organomegaly and renal insufficiency) [8]. Although autoimmune disorders must be excluded in the diagnosis of TAFRO syndrome, it was difficult to explain the anasarca and severe thrombocytopenia as pathophysiological conditions of SS. Therefore, we considered her condition to be one of the primary SS complicated by TAFRO syndrome-like pathophysiology. We initially administered prednisolone at 30 mg/day while she was using furosemide and receiving platelet transfusion, but the platelet count remained low, the volumes of pleural effusion and ascites did not decrease, and the C-reactive protein level was elevated. She was then administered 500 mg methylprednisolone intravenously for 3 days, followed by 50 mg prednisolone daily, and tolvaptan treatment was added. She was also given cyclosporine according to the treatment of TAFRO syndrome. After those therapies were completed, the anasarca began to improve, and administration of tolvaptan was discontinued after 30 days. Because the platelet count remained low, eltrombopag was also administered, after which the platelet count improved. She was discharged on the 76^th^ hospital day, and prednisolone was tapered during the outpatient period. Administration of eltrombopag was stopped at 2 months following her discharge (Figure 3).

## 3. Discussion

Although the central clinical manifestations of SS are sicca features such as dry eyes and mouth, some patients have SS that is complicated by extraglandular manifestations. These include fever, lymphadenopathy, arthritis, and conditions with cutaneous involvement such as annular erythema, interstitial pneumonia, and neurologic manifestations [3]. However, few SS patients are complicated by anasarca and severe thrombocytopenia. Ramos-Casals et al. reported that only 5% of patients with primary SS with pulmonary involvement presented pleural thickening/effusion [10]. Severe thrombocytopenia is also uncommon in SS [11], with Aoki et al. reporting that 7.1% of SS patients were complicated with thrombocytopenia [12].

Histopathological findings of lymph nodes in TAFRO syndrome patients usually show a mixed type of MCD-like features. However, other clinical features of TAFRO syndrome are different from those of MCD and include anasarca and thrombocytopenia [13]. In their proposed diagnostic criteria for TAFRO syndrome published in 2015, Masaki et al. [8] listed diseases to be excluded from TAFRO syndrome, which included malignancies (e.g., lymphoma), autoimmune disorders, and infectious diseases. Our patient fulfilled the diagnostic criteria of TAFRO syndrome, but we were unable to also make a formal diagnosis of TAFRO syndrome because of her SS diagnosis. The continuous low-grade fever and slightly elevated serum IL-6 and VEGF in this patient might also be considered as supportive of an existing pathophysiology of TAFRO syndrome.

A search of the literature yielded five case reports of patients with SS accompanied by clinical features of TAFRO syndrome [14–18]; those cases and ours are summarized in Table 2. Although positivity for the diagnostic features of SS varied, anti-Ro/SS-A antibodies were positive in all six patients. While all of the patients fulfilled the major categories of TAFRO syndrome, only our patient had severe thrombocytopenia that was observed in the early stage. Regarding the minor diagnostic categories in our patient, she did not receive a lymph node biopsy because of the severe thrombocytopenia, and reticulin myelofibrosis was not observed. Sumida proposed a two-step mechanism of SS development: antigen-specific and antigen-nonspecific immune responses. In the antigen-specific phase of the immune response, several factors, including bacterial products, viral products, and autoantigens, are suggested to play an important role in triggering autoimmunity. These antigens are recognized by T cells, which are thereby activated and able to produce various cytokines and induce chronic inflammation, including autoantibodies (e.g., anti-Ro/SS-A and anti-La/SS-B antibodies). Such reactions might lead to the antigen-nonspecific phase [6]. In a report from the Sjögren Big Data Project, anti-Ro/SS-A-positive patients had a lower frequency of sicca symptoms, a higher mean European League against the Rheumatism SS Disease Activity Index (ESSDAI) score, and a higher frequency of activity in the constitutional, cutaneous, renal, hematological, and biological ESSDAI domains compared with anti-Ro/SS-A-negative patients [19]. The presence of positive anti-Ro/SS-A antibodies in all six patients with SS accompanied by TAFRO syndrome-like pathophysiology might indicate a prominent immune reaction of SS that is highly likely to be accompanied by general or extraglandular manifestations. Nevertheless, the pathophysiology of TAFRO syndrome is not fully understood. Elevation of IL-6 and/or VEGF in serum and/or pleural and abdominal effusion was described in 2 cases of multicentric Castleman's disease [20], and slight elevation of IL-6 and VEGF was observed in our patient. Therefore, the activation of the immune system that is induced by SS might also contribute to the induction of TAFRO syndrome-like pathophysiology in these patients. Iwaki et al. reported that the serum level of the inflammatory chemokine interferon-*γ*-induced protein 10 kDa (IP-10) was higher in patients with TAFRO syndrome than those with idiopathic MCD [21], suggesting that IP-10 might be a useful biomarker for characterizing the pathophysiology of TAFRO syndrome. Regarding treatments, all patients received prednisolone and 3 patients including our case were administered cyclosporine. Corticosteroid and IL-6 receptor antagonists are first-line treatments in MCD, while cyclosporine has been reported to be effective for some patients with TAFRO syndrome [22–24]. In cases of SS with TAFRO syndrome-like clinical features, 3 of 6 patients were administered cyclosporine, which was effective in all 3. Cyclosporine selectively inhibits calcineurin, thereby impairing the transcription of the genes that encode IL-2 and several other cytokines in T lymphocytes. We suggest that treatment with cyclosporine in cases of SS with TAFRO syndrome-like features might be advantageous because it suppresses effector T lymphocytes.

In our patient, despite the administration of prednisolone including methylprednisolone pulse therapy, the thrombocytopenia and anasarca did not improve, so cyclosporine was added. To improve anasarca, the vasopressin type-2 antagonist tolvaptan was transiently used with furosemide and was quite effective. However, improvement of thrombocytopenia was delayed. Because platelet-associated immunoglobulin G was positive in this patient, an immune thrombocytopenia-like pathophysiology might have been present, i.e., destruction of platelets through antiplatelet autoantibodies and the reticuloendothelial system and inhibition of platelet maturation by megakaryocytes [25]. Therefore, we added eltrombopag, a thrombopoietin receptor agonist, to the patient's treatment plan. Although the dose was temporarily increased to 37.5 mg/day, eltrombopag therapy was discontinued after 3 months, followed by a reduction in prednisolone with continuation of cyclosporine. Fujimoto et al. reported that eltrombopag was effective in 4 of 5 patients with TAFRO syndrome in a retrospective study in Japan [26]; however, a search on PubMed did not return any individual case reports of administration of eltrombopag for TAFRO syndrome. Although it is necessary to examine whether refractory thrombocytopenia corresponds to all patients with TAFRO syndrome, we propose that administration of eltrombopag might be useful in these patients. Notably, administration of an IL-6 receptor antagonist, tocilizumab, and a human/murine chimeric anti-CD20 antibody, rituximab, were also considered in this patient. However, it was feared that adding each of those agents might cause excessive immunosuppression, given that the patient was already receiving prednisolone and cyclosporine. We, therefore, used eltrombopag as the first choice in this patient and considered tocilizumab or rituximab as the second choice.

In conclusion, we described a patient with primary SS accompanied with clinical features of TAFRO syndrome, refractory anasarca, and thrombocytopenia. Although SS is one of the diseases used to exclude TAFRO syndrome, five other published reports of SS patients also showed the clinical features of TAFRO syndrome [14–18]. The presence of detectable anti-Ro/SS-A antibodies in all six cases suggests that a strong immune response played a role in the induction of TAFRO syndrome-like pathophysiology. When corticosteroid treatment is not effective for TAFRO syndrome, adding cyclosporine might be useful to suppress activation of effector T lymphocytes and reduce the immune response. The results in our case also suggest that tolvaptan for anasarca and eltrombopag for thrombocytopenia could be useful therapeutic agents until the effect of immunosuppressive therapy appears. Experiences from future case reports will be useful to identify additional effective treatments.

## Figures and Tables

**Table 1:** Laboratory findings of the study patient on admission.

WBC (*μ*L)	8,900
Neutrophils (%)	78.5
Eosinophils (%)	0.5
Monocytes (%)	5.5
Lymphocytes (%)	9.5
RBC (10^6^/*μ*L)	3.94
Hb (g/dL)	12.6
Hct (%)	37.5
PLT (10^3^/*μ*L)	27
TP (g/dL)	6.5
Alb (g/dL)	3.0
TB (mg/dL)	0.6
AST (U/L)	18
ALT (U/L)	13
LD (U/L)	175
ALP (U/L)	211
*γ*-GTP (U/L)	44
BUN (mg/dL)	9.3
Creatinine (mg/dL)	0.89
Na (mEq/L)	143
K (mEq/L)	4.3
Cl (mEq/L)	109
CRP (mg/dL)	1.26
CEA (ng/mL)	<0.5
CA19-9 (ng/mL)	<2.0
CA125 (U/mL)	77.3
sIL-2R (U/mL)	745
IgG (mg/dL)	1,643
IgG4 (mg/dL)	38
IgA (mg/dL)	355
IgM (mg/dL)	90
CH50 (U/mL)	60
C3 (mg/dL)	113
C4 (mg/dL)	46.3
RF (IU/mL)	133
ANA (fold)	320
Homogenous (fold)	320
Cytoplasmic	＋
Anti-ds-DNA IgG antibody (IU/mL)	8.3
Anti-Ro/SS-A antibody (fold)	32
Anti-Lo/SS-B antibody (fold)	1
PA-IgG (ng/10^7^)	195
Human herpesvirus 8 DNA	−
IL-6 (pg/mL)	3.01
VEGF (pg/mL)	42.1
<Urinalysis>	
Protein	±
Sugar	−
Blood	−

WBC, white blood cell; RBC, red blood cell; Hb, hemoglobin; Hct, hematocrit; PLT, platelet; TP, total protein; Alb, albumin; TB, total bilirubin; AST, aspartate aminotransferase; ALT, alanine aminotransferase; LD, lactic dehydrogenase; ALP, alkaline phosphatase; *γ*-GTP, *γ*-glutamyl transpeptidase; BUN, blood urea nitrogen; Na, sodium; K, potassium; Cl, chloride; CRP, C-reactive protein; CEA, carcinoembryonic antigen; CA19-9, carbohydrate antigen 19-9; CA125, cancer antigen 125; sIL-2R, soluble interleukin-2 receptor; IgG, immunoglobulin G; IgG4, immunoglobulin G4; IgA, immunoglobulin A; IgM, immunoglobulin M; CH50, 50% hemolytic complement activity; C3, third component of complement; C4, fourth component of complement; RF, rheumatoid factor; ANA, antinuclear antibody; PA-IgG, platelet-associated immunoglobulin G; IL-6, interleukin-6; VEGF, vascular endothelial growth factor.

**Table 2:** Comparison summary of previous cases of Sjögren's syndrome complicated with clinicopathologic features of TAFRO syndrome.

Authors			Iwanaga et al.	Fujimoto et al.	Takasawa et al.	Li et al.	Kojima et al.	Our case
Sjögren's syndrome		Histopathology	+	+	ND	ND	ND	ND
		Oral examination	+	Scintigraphy	ND	ND	ND	+
		Ocular examination	+	+	+	ND	ND	−
		Anti-Ro/SS-A antibody	+	+	+	+	+	+
		Anti-La/SS-B antibody	−	+	−	ND	+	+
TAFRO syndrome	Major categories	Anasarca	+	+	+	+	+	+
		Thrombocytopenia	+	+	+	+	+	+
		Systemic inflammation	+	+	+	+	+	+
	Minor categories	Histology of lymph node biopsy	−	+	+	+	+	ND
		Reticulin myelofibrosis	+	+	+	+	ND	−
		Mild organomegaly	+	+	+	+	+	+
		Renal insufficiency	−	+	+	+	ND	±
Treatments			PSL, CyA	PSL	PSL, CyA	Siltuximab, PSL, RTX, CY, vincristine	PSL	PSL, CyA, eltrombopag

CY, cyclophosphamide; CyA, cyclosporine; PSL, prednisolone; RTX, rituximab; ND, not determined.
